# Editorial: Infection and Inflammation: Potential Triggers of Sudden Infant Deaths

**DOI:** 10.3389/fimmu.2016.00120

**Published:** 2016-03-30

**Authors:** Caroline Blackwell

**Affiliations:** ^1^Immunology and Microbiology, University of Newcastle, New Lambton, NSW, Australia

**Keywords:** sudden infant death, infection inflammation, cytokines, genetics, developmental stage

**The Editorial on the Research Topic**

**Infection and Inflammation: Potential Triggers of Sudden Infant Deaths**

Among infants in industrialized countries, sudden infant death syndrome (SIDS) is still the major cause of death between 1 month and 1 year of age. SIDS is a diagnosis of exclusion, a subset of sudden unexpected deaths in infancy (SUDI), those deaths that remain unexplained following a thorough postmortem, assessment of the medical history, and investigation of the scene of death. The risk factors for SUDI/SIDS identified in epidemiological studies parallel risk factors associated with susceptibility to infection in children, and various studies have identified microorganisms or their products in significant proportions of these deaths (Blackwell et al.). Even in a country, such as Hungary, with a historically low incidence of SUDI/SIDS, there is evidence that infection and inflammation contribute to these deaths (Töro et al.). This collection provides insights into a variety of approaches for the investigation of how genetic, environmental, and developmental risk factors could dysregulate or potentiate the effects of inflammatory responses to “mild” infections that are often reported prior to death of the infant.

Exploration of the role of infectious agents in these deaths has been limited by two factors. The evidence does not fit Koch’s postulates: no single organism has been isolated from all cases of SUDI/SIDS. A variety of organisms, both viruses and bacteria with various virulence factors, has been identified in different studies (Blackwell et al.; Bettelheim and Goldwater; Goldwater). In addition, there is no animal model that accurately reflects all the genetic, developmental, and environmental risk factors associated with SUDI/SIDS. This problem and examples of the models developed are reviewed by Blood-Siegfried. The common thread in the investigation of infection does not appear to be a specific infectious agent but the infant’s inflammatory response to the infection. Therefore, it is important to develop models to assess risk factors that can influence levels of inflammatory mediators, which can affect the major physiological mechanisms proposed to explain SUDI/SIDS – anaphylaxis, poor arousal, hypoxia and apnea, shock, cardiac arrhythmias, hyperthermia, and hypoglycemia.

The differences in the incidence of SIDS among ethnic groups and the excess of male infants indicated that genetic factors might be involved in triggering these deaths. Potential genetic contributions to these deaths and the case for whole exome sequencing are assessed here by Morris. Genetic variations in the immune/inflammatory responses in relation to SUDI are reviewed by Ferrante and Opdal. The potential contribution of two cytokines and polymorphisms in their genes, tumor necrosis factor-α (TNF-α) and interferon-γ (IFN-γ), is explored by Moscovis et al. and Moscovis et al. in relation to these responses to infectious agents and interactions with environmental risk factors such as cigarette smoke. The differences in cytokine responses of male and female fetuses identified by Burns et al. indicate that females might be better able to control inflammatory responses *in utero*.

Two important risk factors for SUDI/SIDS are low birth weight and premature birth. These were explored in relation to two environmental factors that could affect fetal development during pregnancy, exposure to cigarette smoke, and evidence of inflammation. Evidence of inflammation was inversely correlated to gestational age. Exposure to cigarette smoke was also significantly associated with low birth weight and age at delivery. The effects of both evidence of inflammation and exposure to cigarette smoke were more pronounced for male infants (Pringle et al.).

In the absence of a widely accepted model for these infant deaths, their investigation requires the cooperation of a variety of disciplines to attempt to identify why these children have died suddenly. Current evidence indicates that inflammatory responses are dysregulated by combinations of infectious agents and cigarette smoke. Developmental stage of the infant and hormone levels might also affect these responses (Blackwell et al.).

The approaches and techniques developed to examine SUDI/SIDS need to be applied to stillbirths, which have been suggested to be part of the spectrum of infant deaths associated with SIDS (1). While the risk factors are similar, investigations of stillbirths need to take into consideration the responses to infectious agents of both the mother and the infant (Blackwell).

New techniques emerging for studies of infection and inflammation will complement the standard studies carried in routine forensic examinations: assessment of microbiome for both viral and bacterial pathogens; whole genome sequencing of DNA from infants; and screening for a range of inflammatory mediators. These techniques are not usually available for diagnosis of infant deaths. Progress in this area will require cooperation between forensic medicine and pathology services and research groups with the expertise to complement investigations carried out by standard protocols.

## Author Contributions

The author confirms being the sole contributor of this work and approved it for publication.

## Conflict of Interest Statement

The author declares that the research was conducted in the absence of any commercial or financial relationships that could be construed as a potential conflict of interest.
